# Complete genome sequence of *Mycolicibacterium mageritense* strain H4_3_1 isolated from a hybrid biological-inorganic system reactor

**DOI:** 10.1128/MRA.00230-23

**Published:** 2023-10-03

**Authors:** Xiang Feng, Daichi Kazama, Kozo Sato, Hajime Kobayashi

**Affiliations:** 1 Department of Systems Innovation, Graduate School of Engineering, University of Tokyo, Tokyo, Japan; 2 Frontier Research Center for Energy and Resources (FRCER), Graduate School of Engineering, University of Tokyo, Tokyo, Japan; University of Maryland School of Medicine, Baltimore, Maryland, USA

**Keywords:** *Mycolicibacterium*, hydrogen-oxidizing bacteria, hybrid biological-inorganic system

## Abstract

We report the complete genome of *Mycolicibacterium mageritense* strain H4_3_1, which was isolated from a reactor of a hybrid biological-inorganic system. This genome will provide useful information about hydrogen-oxidizing bacteria as well as mycolicibacteria in non-host environments.

## ANNOUNCEMENT

The hybrid biological-inorganic (HBI) system is an emerging variant of bio-electrochemical systems in which hydrogen-oxidizing bacteria (HOB) is used as a biocatalyst to convert CO_2_ into value-added products using H_2_ from water splitting (1). Besides developing electrode materials and operation methodologies (2–4), screening new biocatalysts from various environments is a promising strategy to improve HBI performance (5–7). To this end, an estuarine sediment sample from a coastal river (Imabari, Ehime, Japan: 34.06 N, 132.87 E) was inoculated into an HBI reactor with the minimal medium (7) containing 180 mM phosphate buffer. HOB was enriched in the reactor at 30°C with an applied voltage of 2.0 V, followed by subculturing into a fresh reactor. After two successive subculturing, the reactor medium was serially diluted and spread onto the minimal medium solidified with 1% (wt/vol) gellan gum, followed by incubation under H_2_/CO_2_/O_2_ (80:10:10) at 30°C, resulting in the isolation of *Mycolicibacterium mageritense* strain H4_3_1.

Strain H4_3_1 was propagated in 20 mL of minimal medium under H_2_/CO_2_/O_2_ (80:10:10) at 30°C for 2 days and harvested by centrifugation. Genomic DNA was extracted using MasterPure Gram Positive DNA Purification Kit (Epicentre, Madison, WI, USA) and further purified using a 1.8× AMPureXP (Beckman Coulter, Brea, CA, USA) and DNeasy PowerClean Pro Cleanup Kit (Qiagen, Hilden, Germany). The genomic DNA was sheared into 10–20 kbp fragments using a g-TUBE (Covaris, Woburn, MA, USA). The DNA template library was constructed using SMRTbell gDNA Sample Amplification Kit (PacBio, Menlo Park, CA, USA) and SMRTbell Express Template Prep Kit ver.2.0 (PacBio) according to the manufacturer’s instructions (for library preparation from ultra-low DNA input) and bound to the Sequel ll polymerase 2.2 (PacBio) using Sequel ll Binding Kit 2.2 (PacBio). Sequencing was performed using the Sequel IIe platform (PacBio). SMRT Link (ver.11.0.0.146107) (PacBio) was used to trim overhand adapter sequences from the sequencing reads, and the generated circular consensus sequences with average base quality values <20 were excluded to yield HiFi reads. Default parameters were used for all software unless otherwise noted. The ultra-low PCR adapter sequences were trimmed by using lima (ver.2.6.0) (https://github.com/pacificbiosciences/barcoding). Furthermore, PCR adapters and duplicates were filtered from the HiFi reads using pbmarkdup (ver.1.0.2) (https://github.com/PacificBiosciences/pbmarkdup) and generated a total of 34,035 reads with a mean size of 7,316 bp. Short HiFi reads (below 1,000 bp) were excluded using Filtlong (ver.0.2.1) (https://github.com/rrwick/Filtlong). The remaining 30,128 HiFi reads were assembled using Flye (ver.2.9.1-b1780) (8). The resulting genome of strain H4_3_1 comprised one single contig of 7,607,618 bp sequence (G+C content 67.1%), with coverage of 33×. The assembly graph was visualized by Bandage (ver.0.8.1) (https://github.com/rrwick/Bandage) (9), indicating that the assembled genome is circular. Genome annotation was performed using Prokka (ver.1.14.5) (https://github.com/tseemann/prokka) (10). Of the 7,441 predicted genes, 7,330 were protein-coding genes and 71 were RNAs (six rRNAs, 64 tRNAs, and one tmRNA). *In silico* DNA-DNA hybridization (DDH) analysis using the Genome-to-Genome Distance Calculator (3.0, https://ggdc.dsmz.de) (11) showed the closest DDH value of 92.4% toward *M. mageritense* CIP 104973 (GCA_000612825), indicating the strain H4_3_1 is a member of *M. mageritense*.

## Data Availability

The genome project is indexed at the DNA Data Bank of Japan (DDBJ) under the BioProject accession number PRJDB15418. The complete genome sequence for *M. mageritense* strain H4_3_1 is available in the DDBJ/EMBL/GenBank databases under the accession number AP027452. Raw reads can be found under Sequence Read Archive (SRA) with accession number DRR452424.
